# Antibody to Marinobufagenin Reverses Placenta-Induced Fibrosis of Umbilical Arteries in Preeclampsia

**DOI:** 10.3390/ijms19082377

**Published:** 2018-08-13

**Authors:** Olga V. Fedorova, Valentina V. Ishkaraeva, Yulia N. Grigorova, Vitaly A. Reznik, Nikolai I. Kolodkin, Irina E. Zazerskaya, Valentina Zernetkina, Natalia I. Agalakova, Natalia I. Tapilskaya, C. David Adair, Edward G. Lakatta, Alexei Y. Bagrov

**Affiliations:** 1 Laboratory of Cardiovascular Science, National Institute on Aging, NIH, Baltimore, MD 21224, USA; fedorovo@mail.nih.gov (O.V.F.); yulia.grigorova2@nih.gov (Y.N.G.); valentina.zernetkina@nih.gov (V.Z.); lakattae@grc.nia.nih.gov (E.G.L.); 2 Institute of Neonatology, Almazov Federal Heart, Blood and Endocrinology Center, St. Petersburg 197431, Russia; yahont84@list.ru (V.V.I.); zazera@mail.ru (I.E.Z.); 3 Department of Obstetrics and Gynecology, School of Pediatric Medicine, St. Petersburg 194353, Russia; vitaliy-reznik@mail.ru; 4 Institute of Highly Pure Biopreparations, St. Petersburg 197110, Russia; kolodkin@hpb-spb.com; 5 Sechenov Institute of Evolutionary Physiology and Biochemistry, 44 Torez Prospect, St. Petersburg 194223, Russia; nagalak@mail.ru; 6 DO Ott Institute of Obstetrics and Gynecology, St. Petersburg 199034, Russia; tapnatalia@yandex.ru; 7 Department of Obstetrics and Gynecology, University of Tennessee, Chattanooga, TN 37403, USA; Adair@rocob.com

**Keywords:** preeclampsia, Na/K-ATPase, cardiotonic steroids, digitalis-like factors, marinobufagenin, immunotherapy, fibrosis, collagen, Fli-1

## Abstract

Background: Previous studies implicated cardiotonic steroids, including Na/K-ATPase inhibitor marinobufagenin (MBG), in the pathogenesis of preeclampsia (PE). Immunoneutralization of heightened MBG by Digibind, a digoxin antibody, reduces blood pressure (BP) in patients with PE, and anti-MBG monoclonal antibody lessens BP in a rat model of PE. Recently, we demonstrated that MBG induces fibrosis in cardiovascular tissues via a mechanism involving inhibition of Fli-1, a nuclear transcription factor and a negative regulator of collagen-1 synthesis. Objectives and Methods: We hypothesized that in PE, elevated placental MBG levels are associated with development of fibrosis in umbilical arteries. Eleven patients with PE (mean BP 124 ± 4 mmHg; age 29 ± 2 years; 39 weeks gest. age) and 10 gestational age-matched normal pregnant subjects (mean BP 92 ± 2 mmHg; controls) were enrolled in the clinical study. Results: PE was associated with a higher placental (0.04 ± 0.01 vs. 0.49 ± 0.11 pmol/g; *p* < 0.01) and plasma MBG (0.5 ± 0.1 vs. 1.6 ± 0.5 nmol/L; *p* < 0.01), lower Na/K-ATPase activity in erythrocytes (2.7 ± 0.2 vs. 1.5 ± 0.2 µmol Pi/mL/hr; *p* < 0.01), 9-fold decrease of Fli-1 level and 2.5-fold increase of collagen-1 in placentae (*p* < 0.01) vs. control. Incubation of umbilical arteries from control patients with 1 nmol/L MBG was associated with four-fold decrease in Fli-1 level and two-fold increase in collagen-1 level vs. those incubated with placebo (*p* < 0.01), i.e., physiological concentration of MBG mimicked effect of PE in vitro. Collagen-1 abundance in umbilical arteries from PE patients was 4-fold higher than in control arteries, and this PE-associated fibrosis was reversed by monoclonal anti-MBG antibody ex vivo. Conclusion: These results demonstrate that elevated placental MBG level is implicated in the development of fibrosis of the placenta and umbilical arteries in PE.

## 1. Introduction

Preeclampsia (PE) represents one of the most serious complications of pregnancy, leading to maternal and fetal morbidity and mortality. The mechanisms of its pathogenesis are not well understood, and there is no effective treatment of this obstetric malady [1]. Graves et al. were the first to demonstrate increased levels of cardiotonic steroids (CTS) in pregnancy and to hypothesize that CTS are involved in the pathogenesis of pregnancy-induced hypertension, including PE [2]. Marinobufagenin (MBG), a bufadienolide CTS, is an inhibitor of Na/K-ATPase with has a higher affinity to the alpha1 isoform, a major isoform in vascular wall and the exclusive isoform in the kidney [3,4]. Pregnancy is associated with fluid and sodium retention, which are the stimuli for MBG production [5]. Recently MBG was identified in non-pregnant human plasma and in plasma from pregnant woman utilizing a LC-MS assay [6]. In normal pregnancy, however, MBG levels are not high enough to produce vasoconstriction [7]. Notably that in patients with PE, elevation of arterial blood pressure (BP) is associated with markedly increased plasma levels of MBG, which are significantly higher than in normal pregnancy [5,8,9], and lower Na/K-ATPase activity in erythrocytes from PE patients compared to non-complicated pregnancy [8,9,10,11]. In a rodent model, pregnancy is associated with the development of salt-sensitivity, thus, pregnant rats on a high salt intake exhibited higher arterial BP vs. virgin female rats [7]. In vivo administration of anti-MBG antibody to pregnant NaCl-supplemented rats lowers arterial BP and is associated with an increase in the vascular sodium pump activity [9]. 

More recently MBG was implicated in pathogenesis of PE by induction of vascular fibrosis [12,13]. MBG acted upstream of Fli-1, a negative collagen-1 regulator, causing its reduction which resulted in activation of collagen-1 [12]. In a previous study we demonstrated that MBG is synthesized by human trophoblast cells via an extra-hepatic “acidic” bile acid pathway [14]. However, it remained unknown (i) whether the placenta is a site of MBG production in PE, (ii) whether MBG can stimulate fibrosis in control umbilical arteries ex vivo, and (iii) whether fibrosis of PE umbilical arteries could be treated with antibody to MBG. 

## 2. Results

Eleven patients with PE (age, 28 ± 2 years; gestational age, 36 ± 1 weeks) and 19 normotensive pregnant subjects (age, 26 ± 1 years; gestational age, 37 ± 1 weeks) were enrolled in the study (Table 1). Patients with PE demonstrated an increase in systolic and diastolic blood pressures which was accompanied by elevated levels of MBG in placentae and plasma (Table 1). In the patients with PE, elevated levels of MBG were associated with a substantial inhibition of Na/K-ATPase in the erythrocytes, as compared to that in subjects with uncomplicated pregnancies (Table 1). 

**Figure 1:**
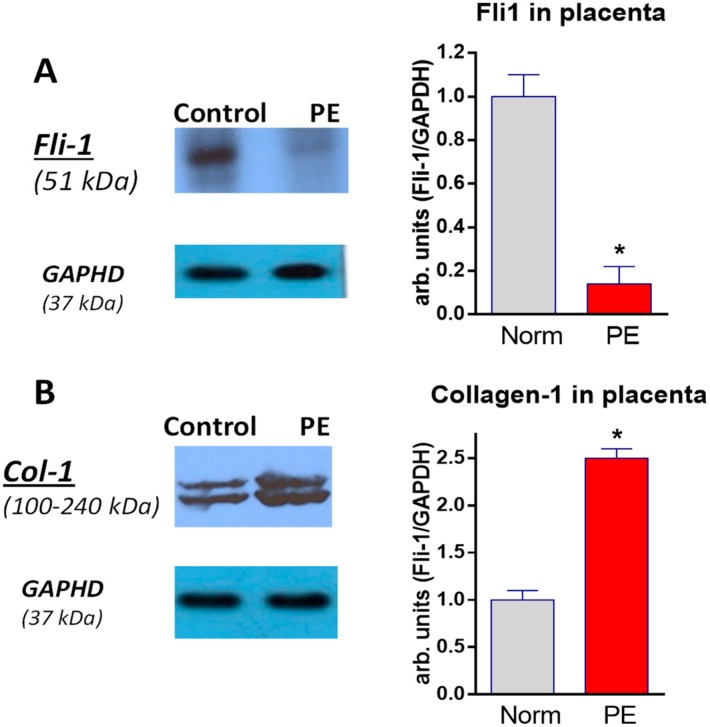
Western blots of Fli-1 (**A**) and collagen-1 (**B**) in placentae from normotensive pregnant subjects (grey columns) and patients with preeclampsia (PE; red columns). Left—representative Western blots, right—bars representing means ± SEM from 4 densitometry measurements. By *t*-test: * *p* < 0.01 vs. normotensive pregnant subjects. Levels of Fli-1 and of collagen-1 were normalized against levels of GAPDH.

As presented in Figure 1A, development of PE was associated with a 9-fold reduction in the expression of placental Fli-1, and, conversely, level of collagen-1 in the placentae (Figure 1B) was significantly elevated vs. tissue from normal pregnancy.

**Figure 2:**
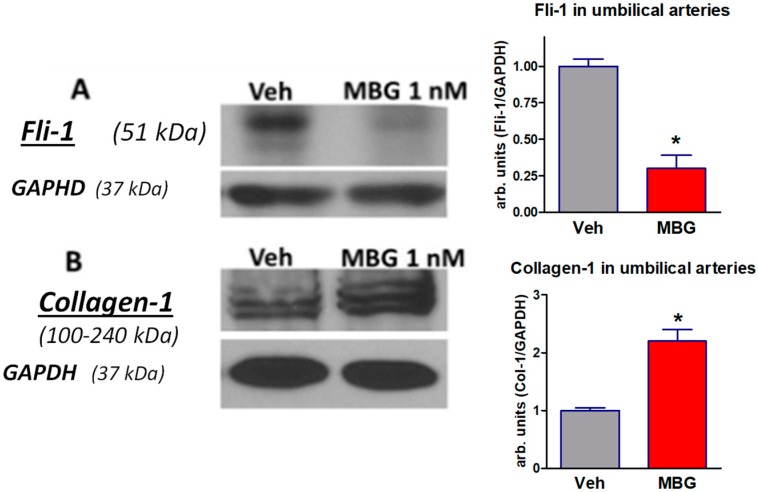
Western blots of Fli-1 (**A**) and collagen-1 (**B**) in placentae from normotensive pregnant subjects incubated with vehicle (grey columns) or 1 nM MBG (red columns). Left—representative Western blots, right—bars representing means ± SEM from 4 densitometry measurements. By *t*-test: * *p* < 0.01 vs. vehicle. Levels of Fli-1 and of collagen-1 were normalized against levels of GAPDH.

Next, we studied whether ex vivo incubation of explants of placentae and umbilical arteries from subjects with non-complicated pregnancies in the presence of nanomolar concentrations of MBG would mimic effects of PE. Incubation of umbilical arteries explants from control patients with 1 nmol/L MBG for 24-h was associated with four-fold decrease in Fli-1 level and two-fold increase in collagen-1 level vs. those incubated with placebo (Figure 2), i.e., physiological concentration of MBG mimicked effect of PE in vitro.

**Figure 3:**
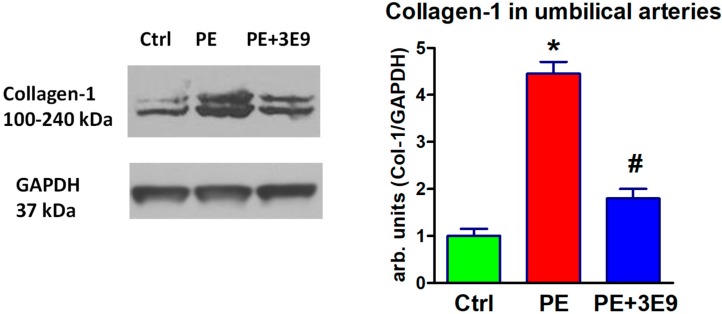
Western blots of collagen-1 in umbilical arteries from normotensive pregnant control subjects (green column; Ctrl), patients with PE (red column, PE), and patients with PE incubated with 3E9 anti-marinobufagenin antibody (blue column, PE + 3E9). Left, representative Western blots; right, bars representing means ± SEM from 4 densitometry measurements. By 1-way ANOVA followed by Neuman-Keuls test: * *p* < 0.01 vs. Ctrl; # *p* < 0.05 vs. PE. Levels of collagen-1 were normalized against levels of GAPDH.

Collagen-1 abundance in umbilical arteries from PE patients was 4-fold higher than in control arteries, and PE-associated fibrosis was reversed by preincubation the arteries with monoclonal anti-MBG antibody ex vivo for 24 h (Figure 3).

## 3. Discussion

PE represents one of the most serious complications of pregnancy, leading to maternal and fetal morbidity and mortality. The mechanisms of PE pathogenesis are not well understood, and there is no effective cure or prophylaxis of this obstetric malady [1]. MBG is one of the factors, implicated in pathogenesis of PE via induction of vasoconstriction and vascular fibrosis [2,14]. In the present study we, for the first time, demonstrate that (i) fibrosis in placenta and umbilical arteries in PE patients is accompanied by elevated placental MBG and a dramatic decrease in Fli-1 level, and (ii) that antibody against MBG block PE-induced fibrosis in the umbilical arteries ex vivo. These results provide evidence that PE manifests itself not only as a condition associated with blood pressure elevation, and that classic “ionic mechanism” of CTS action does not fully describe the pathogenesis of PE [15]. Recent discovery of signaling functions of the sodium pump added a new dimension to the studies of CTS and fibrosis, i.e., demonstrated the significance of the “signaling mechanism” of the Na/K-ATPase [16]. 

In the present study, the placental levels of Fli-1 were dramatically lower, while collagen-1 levels were higher in both placenta and umbilical arteries in PE compared to the tissues from control non-complicated pregnancy. Notably that the treatment of the umbilical artery explants from PE patients with anti-MBG mAb was accompanied by significant decrease in collagen-1 abundance. Inhibition of Fli-1, a nuclear transcription factor and a member of the ETS family is implicated in MBG-induced fibrosis [17]. Fli-1 acts as a negative regulator of collagen-1 synthesis and it competes with another transcription factor, ETS-1, to maintain a balance between stimulation and repression of *Col1a2* gene [18]. The binding of MBG converts Na/K-ATPase to a signal transducer that complexes with Src and the epidermal growth factor receptor (EGFR). The Na/K-ATPase/Src/EGFR complex initiates a signal cascade, which activates phospholipase C (PLC) resulting in phosphorylation of PKCδ and its translocation to the nucleus. In the nucleus, phosphorylated PKCδ phosphorylates Fli-1, which leads to more rapid catabolism of Fli-1, a negative regulator of collagen synthesis, and removal of Fli-1 inhibition on the collagen-1 promoter and thus increases in procollagen expression and collagen production [12,17]. U-73122, a PLC antagonist, prevented an increase in nuclear PKCδ, whereas rottlerin (a PKC inhibitor) prevented MBG-induced increase in procollagen expression supporting this concept [17]. However, it remains unknown whether the Fli-1 signaling is generated by the fibroblasts or by a variety of cells. Recently we have demonstrated that the stimulation of the cultured rat vascular smooth muscle cells with MBG resulted in the up-regulation of collagen-1 production [19], and that the pro-fibrotic Fli-1 signaling was activated by MBG and the increased collagen-1 in the freshly prepared rat cardiac myocytes (Zhang et al., unpublished, 2018). The study on the cellular specificity of Fli-1-dependent signaling in the placenta and the umbilical vasculature and surrounding tissues merits future investigations.

Previously, we demonstrated that the development of cardiac fibrosis in the presence of elevated MBG levels was associated with reduction of Fli-1 in uremic rats, and that a single injection of anti-MBG antibody markedly reduced cardiac levels of collagen-1 in with an increase in the levels of Fli-1 in this rodent model [18]. Our present finding of the antifibrotic effect of antibody to MBG in PE patients is in agreement with the previous observations made in a rat model of uremic cardiomyopathy [20]. 

In conclusion, development of fibrosis in placentae and umbilical arteries in PE patients could be attributed to the pro-fibrotic activity of an endogenous Na/K-ATPase inhibitor, MBG. For the first time, we demonstrated that MBG-induced suppression of Fli-1 and vascular fibrosis in PE is reversible by an antibody against MBG. 

## 4. Methods

### 4.1. General

The protocol for this study was approved by the Ethical Committee of Almazov Federal Heart, Blood and Endocrinology Center, St. Petersburg, Russia, and by the Institutional Review Board of Medstar Research Institute, Washington, DC (Marinobufagenin as a Target for DIGIBIND in Preeclampsia # 2006–222, 8 November 2012). Written informed consent was obtained from all participants. Eleven participants who were admitted were enrolled in the study after giving informed consent. Diagnosis of PE was based on the criteria of the American Congress of Obstetrics and Gynecology [21]. This definition includes systolic blood pressure of at least 140 mmHg, or diastolic blood pressure of at least 90 mmHg, and new onset proteinuria (urinary protein excretion more than 0.3 g/24 h or a urinary protein concentration of more than 1 g/L in at least two random urine specimens collected 6 h or more apart in a pregnancy after the 20th week of gestation. Exclusion criteria are: a clinical need for digitalis drugs, antecedent history of essential hypertension, and chronic cardiovascular, renal, hepatic, or endocrine disorders, the MgSO_4_ therapy [11]. In addition, 10 age-matched and gestational age-matched normotensive subjects with uncomplicated pregnancies were enrolled to the study to serve as the control group.

### 4.2. Immunoassay

MBG assay is based on the competition between immobilized antigen (MBG-glycoside-thyroglobulin) and MBG, other cross-reactants, or endogenous CTS within the sample for a limited number of binding sites on an anti-MBG mAbs. Secondary (goat anti-mouse) antibody labeled with nonradioactive Europium was obtained from Perkin-Elmer (Waltham, MA, USA) [9]. The sensitivity of this MBG DELFIA is 0.05 nmol/L, and the cross-reactivity of 4G4 mAb used in this assay with other steroids is (%): MBG—100; marinobufotoxin—43; cinobufotalin—40; telocinobufagin—14; resibufagenin—0.5; bufalin—0.08; cinobufagin—0.07; digoxin—0.03; ouabain—0.005; digoxigenin—0.004; proscillaridin A, digitoxin, aldosterone, progesterone, prednisone, corticosterone, and thyroglobulin <0.001.

### 4.3. Na/K-ATPase Measurement

Six mL blood was used for the measurement of erythrocyte Na/K-ATPase activity as reported previously in detail [9]. Erythrocytes were washed three times in an isotonic medium (145 mmol/L NaCl in 20 mmol/L Tris buffer, pH 7.6 at 4 °C). Erythrocytes were preincubated with Tween-20 (0.5%) in sucrose (250 mmol/L) and Tris buffer (20 mmol/L; pH 7.4, 37 °C) for 30 min, and were incubated for 30 min in the medium (mmol/L): Na 100, K 10, MgCl_2_ 3, EDTA 0.5, Tris 50, ATP 2 (pH 7.4, 37 °C) in the final dilution 1:40. The reaction was stopped by the addition of trichloracetic acid to final concentration 7%. Total ATPase activity was measured by the production of inorganic phosphate (P_i_), and Na/K-ATPase activity was estimated by the difference between ATPase activity in the presence and in the absence of 5 mmol/L ouabain. 

### 4.4. Placentae

Placentae were perfused with a solution containing (in mmol/L) NaCl 120; KCl 4; CaCl_2_ 2.5; MgCl_2_ 2.0; NaH_2_PO_4_ 1.1; NaHCO_3_ 24; and glucose 5.6 (pH 7.4) until complete removal of blood was accomplished, the tissue was minced and homogenized [22]. Homogenates were divided into two parts one of which was immediately frozen for determination of angiogenic factors by Western blotting (below), and another used for measurement of MBG. For MBG measurement, the homogenate was extracted with chloroform and dried under a vacuum. The dried extract was sonicated in water (1:5 *w*/*v*) and applied on a reverse-phase C-18 SepPak ‘long body’ cartridge, eluted with 80% acetonitrile, and dried in a SpeedVac centrifuge (Savant, Hicksville, New York, NY, USA). Levels of MBG were determined in placental extracts as described below (Immunoassays). The placentas pieces were homogenized in RIPA lysis buffer (Santa Cruz Biotechnology, Inc., Santa Cruz, CA, USA) and centrifuged at 10,000 *g* for 30 min at 4 °C to remove tissue debris. Solubilized proteins were used for Western blotting analysis (below). 

### 4.5. Umbilical Arteries

After the delivery, umbilical arteries were separated from surrounding tissues and either immediately tested for contractile/relaxant properties (below) or processed for determination of Fli-1 and collagen-1 abundance by Western blotting analysis [13] (below). Umbilical arteries from subjects with uncomplicated pregnancies were also treated ex vivo with MBG to mimic effects of preeclampsia. Explants of umbilical arteries from subjects with uncomplicated pregnancies were placed in Dulbecco’s Modified Eagle Medium supplemented with high glucose, glutamine, pyrodoxin hydrochloride, and sodium pyruvate (25 mg/kg gentamicin) (Invitrogen, Carlsbad, CA, USA). Arterial explants were incubated for 24 h in a 5% CO_2_ atmosphere at 37 °C in the presence of MBG (1 nmol/L) or vehicle (control). Both MBG/vehicle-treated vascular rings were used for preparation of homogenates (below). Arterial rings were minced by scissors, homogenized in RIPA lysis buffer (Santa Cruz Biotechnology, Inc., Dallas, TX, USA) and centrifuged at 10,000 *g* for 30 min at 4 °C to remove tissue debris. Solubilized proteins were used for Western blotting analysis (below).

### 4.6. Western Blotting Analysis

Abundance of Fli-1 (rabbit polyclonal antibody; Santa-Cruz Biotechnology; 1:500) and collagen-1 (goat polyclonal antibody, Southern Biotechnology, Birmingham, AL, USA; 1:200) was determined in placental membranes and membranes from the umbilical arteries. Solubilized proteins were separated by 4–12% Tris-Glycine polyacrylamide gel electrophoresis, were transferred to a nitrocellulose membrane and were visualized using the above specific antibodies followed by incubation with peroxidase-conjugated anti-rabbit antiserum (GE Health Care/Life Sciences, Pittsburgh, PA, USA, 1:1000), or anti-goat antiserum (Santa Cruz Biotechnology Inc., 1:1000) [13]. Bands were visualized by 1–15 min exposure of nitrocellulose membrane on Kodak SAR5 film. To normalize levels of Fli-1 and collagen-1 against levels of glyceraldehydes-3-phosphate dehydrogenase (GAPDH) membranes were stripped and re-probed with a mouse monoclonal antibody against GAPDH (GE Health Care/Life Sciences, 1:4000) followed by anti-mouse peroxidase-conjugated antiserum (GE Health Care/Life Sciences, 1:1000).

### 4.7. Statistical Analysis

The results are presented as mean ± SEM. Data were analyzed using 1-way analysis of variance (ANOVA) (intergroup analysis) or by repeated measures ANOVA (intragroup analysis) followed by Newman-Keuls test, and by two-tailed *t*-test when applicable (Graph Pad Prism Software, San Diego, CA, USA). A two-sided *p* value of less than 0.05 was considered to be statistically significant.

## Figures and Tables

**Table 1:** Characteristics of the study participants.

	Control Group (*n* = 10)	Patients with PE (*n* = 11)
Age (years)	28 ± 2	29 ± 2
Gestational age (weeks)	39.0 ± 0.4	39.0 ± 0.5
Systolic BP (mm Hg)	112 ± 3	157 ± 5 *
Diastolic BP (mm Hg)	72 ± 2	94 ± 2 *
Protein excretion (g per 24 h)	*n*/d	2.12 ± 0.46 *
Plasma MBG (nmol/L)	0.47 ± 0.10	1.6 ±0.5 *
Placental MBG (pmol/g)	0.04 ± 0.01	0.49 ± 0.11 *
Erythrocyte Na/K-ATPase (µmol Pi/mL/h)	2.7± 0.2	1.5 ± 0.2 *

Means ± SEM. By two-tailed *t*-test: * *p* < 0.01 vs. control group. Control group, subjects with normal pregnancy; PE, patients with preeclampsia; BP, blood pressure; MBG, marinobufagenin.
